# Elevation of High-Mobility Group Protein Box-1 in Serum Correlates with Severity of Acute Intracerebral Hemorrhage

**DOI:** 10.1155/2010/142458

**Published:** 2010-09-29

**Authors:** Yu Zhou, Kun-Lin Xiong, Sen Lin, Qi Zhong, Feng-Lin Lu, Hong Liang, Jing-Cheng Li, Jing-Zhou Wang, Qing-Wu Yang

**Affiliations:** ^1^Department of Neurology, Daping Hospital, The Third Military Medical University, Changjiang Branch Road no. 10, Yuzhong District, Chongqing 400042, China; ^2^Department of Radiology, Daping Hospital, The Third Military Medical University, Yuzhong District, Chongqing 400042, China; ^3^College of Biomedical Engineering, Chongqing University, Chongqing 400044, China; ^4^Department of Neurosurgery, Daping Hospital, The Third Military Medical University, Yuzhong District, Chongqing 400042, China

## Abstract

High-mobility group protein box-1 (HMGB1) is a proinflammatory involved in many inflammatory diseases. However, its roles in intracerebral hemorrhage (ICH) remain unknown. The purpose of this study was to examine the correlation between changes in serum levels of HMGB1 following acute ICH and the severity of stroke as well as the underlying mechanism. Changes in serum levels of HMGB1 in 60 consecutive patients with primary hemispheric ICH within 12 hours of onset of symptoms were determined. The correlation of HMGB1 with disease severity, IL-6, and TNF-*α* was analyzed. Changes in HMGB1 levels were detected with ELISA and Western blot. Compared with normal controls, patients with ICH had markedly elevated levels of HMGB1, which was significantly correlated with the levels of IL-6 and TNF-*α*, NIHSS score at the 10th day, and mRS score at 3 months. In comparison with the control group, the levels of HMGB1 in the perihematomal tissue in mice with ICH increased dramatically, peaked at 72 hours, and decreased at 5 days. Meanwhile, heme could stimulate cultured microglia to release large amounts of HMGB1 whereas Fe^2+/3+^ ions failed to stimulate HMGB1 production from microglia. Our findings suggest that HMGB1 may play an essential role in the ICH-caused inflammatory injury.

## 1. Introduction

A growing body of evidence has shown that inflammatory responses play an important role in ICH-induced injury [1–3]. Hematoma components and metabolites of red blood cells in ICH can provoke brain tissues to produce inflammatory response, causing cerebral edema and aggravating neurological deficits [4]. In addition, ICH can also elicit strong systemic inflammatory responses, resulting in abundant release of inflammatory factors such as IL-6 and TNF-*α*, further exacerbating neurological deficits [2]. However, the mechanism responsible for ICH-induced inflammatory responses has not been fully understood.

HMGB1 is a nuclear factor and a DNA-binding protein [5–7]. Recently, an increasing number of studies have shown that it is also a proinflammatory factor capable of stimulating the production of other inflammatory factors and plays an important role in sepsis and cerebral ischemia-caused inflammatory injury [8–10]. However, its role in inflammatory injury resulting from ICH remains largely undefined. A previous study [11] showed that levels of HMGB1 in the peripheral blood and cerebrospinal fluid (CSF) in subarachnoid hemorrhage (SAH) increased significantly and were positively associated with disease severity. Similarly, our previous study [12] showed that the expression of Toll-like receptor 4 (TLR4), a receptor of HMGB1, in the perihematomal tissue in ICH was significantly elevated and exhibited a significant positive correlation with disease severity. Therefore, we speculate that serum levels of HMGB1 may also increase dramatically following ICH. The aim of the present study was to examine the association between changes in serum levels of HMGB1 following acute ICH and stroke severity and investigate the underlying mechanism, in an attempt to shed light on the role of HMGB1 in ICH-caused inflammatory injury.

## 2. Materials and Methods

### 2.1. Subjects

This prospective study included 65 patients with acute ICH admitted to the Stroke Center of our hospital within 12 hours of symptom onset (mean: 7.4 hours) from January 2009 to March 2010. Patient selection was performed based on a previous study [13] and the criteria recommended by the Forth Chinese National Meeting for Cerebrovascular Disease (1996). Exclusion criteria were as follows: (1) patient age <18 years; (2) patients who underwent surgery after admission; (3) patients who were in coma or died on admission or 48 hours after admission; (4) patients with hemorrhage due to brain tumor, trauma, drug abuse, coagulation abnormalities, anticoagulation therapy, or vascular malformations; (5) obvious inflammatory conditions (e.g., infectious disease, systemic lupus erythematosus, rheumatism, and rheumatoid disease) within the half year before enrolment; hospital-acquired infection (confirmed by the patient's symptoms and laboratory findings); acute myocardial infarction (diagnosed according to the patient's symptoms and electrocardiographic findings); acute ischemia of liver (confirmed by liver function test); autoimmune disease (diagnosed according to the patient's symptoms and positive autoimmune antibodies); patients who took glucocorticosteroids or immunodepressants; patients who were incompliant to the study protocol or could not undergo all the tests required by the study. A total of five patients were excluded from the study, including three patients who died and two who were lost to follow-up. The remaining 60 patients with acute ICH were included for analysis.

A total of 41 healthy people who underwent regular physical examinations at our hospital were included in the control group. The controls were matched with the patients in terms of age and gender, and they did not have acute ischemia of the heart, peripheral tissues, or brain within 12 months before enrolment (see Table 1). Signed informed consent was obtained from all the study subjects, and the study protocol was approved by the Medical Ethics Committee of the hospital. Peripheral leukocytes and platelets were counted.

All patients were admitted at an acute stroke unit and treated according to the guidelines of the European Stroke Initiative [14]. The patients underwent head CT examinations on admission, 72 hours and 7 days after admission, respectively. Size of hematoma and peripheral hypodensity volume of ICH were measured using the formula of the perpheral, and edema volume was calculated by subtracting the volume of the ICH from that of the total lesion. Mass effect was considered when ventricular asymmetry or shifting of the midline structure was observed. All CT scans were centrally evaluated by an investigator who was masked to clinical data.

The National Institutes of Healthy Stroke Scale (NIHSS) was used to rate the severity of stroke [15], and modified Rankin Scale (mRS) scores were used to assess the functional status of patients at 3 months after the onset of stroke, and poor outcome was defined as modified rankin scale score >2.

A total of 10 mL of blood sample was drawn from the median cubital vein on admission. Serum were isolated at room temperature. Serum samples were stored −80°C before measurement.

### 2.2. HMGB1 ELISA Assay

An ELISA kit for human HMGB1 assay (R&D Systems Inc. MN, USA) was used to determine the concentration of HMGB1. The detection threshold of this assay is <1 ng/mL. The between-assay coefficient of variations is <10%. Serum levels of TNF-*α* and IL-6 were determined by ELISA according to the instructions provided with the kits (Beijing Jingmei Co. China).

### 2.3. HMGB1 Western Blot Assay

Proteins were prepared from serum and perihematomal brain tissue. Briefly, proteins were separated by SDS-polyacrylamide gel electrophoresis and transferred onto Hybond ECL membranes (Amersham Pharmacia). The ECL membranes were incubated with the primary antibodies including mouse antihuman HMGB1 (for HMGB1 assay in serum, 1 : 1000, Santa Cruz) or rabbit antimouse HMGB1 (for HMGB1 assay in brain tissue, 1 : 5000, Santa Cruz), followed by incubation with peroxidase-conjugated secondary antibodies (1 : 2000, Jingmei, China). The signals were detected with ECL system (Amersham Pharmacia). The same membranes were probed with antibody for glyceraldehyde-3-phosphate dehydrogenase (GAPDH) after being washed with stripping buffer. The signals were quantified by scanning densitometry and computer-assisted image analysis.

### 2.4. Mouse ICH Model

C57BL/6 mice (male, 10 weeks old, weighting 20 to 25 g) in our laboratory were employed with ICH model. The ICH model was established in mice according to our previous study and a previously described method [12, 16]. Briefly, anesthesia was induced in mice by intraperitoneal injection of chloral hydrate (40 mg/kg). Subsequently, 25 *μ*l of non-anticoagulated blood was drawn from the tail vein and injected into the right caudate nucleus and putamen under the guidance of a stereotactic device (Stoelting Co., US). The sham-operated group was injected 25 *μ*l of normal saline (0.9%). The needle hole left by the procedure was sealed with bone wax and the incision was sutured. Mice were randomized into ICH group (*n* = 15) and sham-operated group (*n* = 15). After successful modeling, the mice were sacrificed by cervical dislocation at 24 hours, 48 hours, 72 hours, and 5 days. One hundred mg of perihematomal brain tissue were collected and stored at −80°C. A portion of the tissue sample was homogenized, and the resultant supernatant was detected with ELISA assays. Another portion of the stored sample was used for protein extraction and subsequent Western blotting.

### 2.5. Microglia Culture and Stimulation

For primary microglial culture, cells were dissociated from the cerebral hemispheres of 1- to 2-days postnatal mice (C57BL/c mice, 10 weeks old, weighting 20 to 25 g) brains and seeded into a 6-well plate at a density of 1 × 10^6^/mL with DMEM (Sigma, St. Louis, MO) containing 10% FBS (Hyclone, Logan, UT). Culture media were changed twice per week for 2 weeks, and then microglia were detached by mild shaking and filtered through a nylon mesh to remove astrocytes. After centrifugation at 1000 × g for 10 minutes, the cells were resuspended in a fresh DMEM supplemented with 10% FBS and plated at a final density of 4 × 10^4^/mL cells on a 24-well culture plate. The following day, cells were subjected to the experiments. The microglial cultures used were >95% pure.

Microglia cultures were stimulated with various concentrations of heme (0–30 *μ*M), FeCl_3_ and FeSO_4_ (30 *μ*M) for various time periods according to a method previously described [17]. After discarding the culture supernatant, fresh culture medium was added to the microglia. The cells were cultured for another 12 hours before collection of the supernatant for HMGB1 determination.

### 2.6. Statistical Analysis

Continuous variables were expressed as mean ± (SD) or median and quartiles and compared by Student's *t*- or the Mann-Whitney tests as appropriate. The categorical variables were reported as counts and percentages and analyzed with the *χ* ^2^ test. Spearman's correlation coefficients were computed for evaluating associations among parameters: HMGB1, NIHSS scores, mRS scores, IL-6, and TNF-*α*. SPSS11.5 software was used in all statistical analyses. Values of *P* < .05 were considered statistically significant in all tests.

## 3. Results

### 3.1. Serum Levels of HMGB1 in Patients with Acute ICH Increased Markedly and Positively Correlated with the Severity of Stroke, IL-6, and TNF-*α*

**Figure 1:**
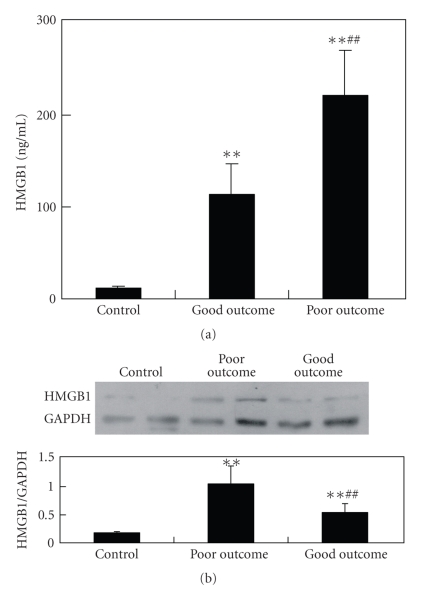
Serum level of HMGB1 increased significantly in patients with acute ICH. Data are presented as mean ± standard deviation. (a) ELISA assay of the changes of HMGB1 serum levels in patients. (b) A representative picture of Western blot assay showing the changes of HMGB1 serum levels in patients. GAPDH was chosen as the internal control. The changes of HMGB1 were expressed as the ratio of the optical density values of HMGB1 band to the optical density values of GAPDH band. **versus control; *P* < .01; ^##^versus good outcome group; *P* < .01.

The demographic data, clinical, biochemical, and imaging features of the patients are presented in Table 1. An unfavorable outcome was observed in 48.3% (29/60) of the patients. Higher stroke severity, larger hematoma volume, larger edema volume, intraventricular extension, mass effect, and higher baseline IL-6 and TNF-*α* were associated with poor clinical outcome. Compared with the control group, the serum levels of HMGB1 increased markedly in patients with acute ICH. Significant difference regarding HMGB1 serum levels was noted among patients with different outcomes. Patients with a poor outcome had significantly higher serum levels of HMGB1 than patients with a favorable outcome (221.4 ± 49.5 versus 114.6 ± 32.6; *P* < .001). As shown in Figure 1, the higher the levels of HMGB1, the worse the outcome a patient would have. Correlation analysis showed that the baseline level of HMGB1 was significantly positively correlated with the baseline levels of IL-6 (*r* = 0.732; *P* < .001) and TNF-a (*r* = 0.620; *P* < .01). Moreover, the correlation analysis also revealed that the baseline level of HMGB1 was significantly positively correlated with NIHSS score at 10 days (*r* = 0.845; *P* < .001) and mRS score at 3 months (*r* = 0.776; *P* < .0001). These results show that the elevation of HMGB1 serum levels in patients with acute ICH is significantly positively correlated with the severity of stroke.

### 3.2. HMGB1 Levels in the Perihematomal Brain Tissue in Mice with ICH were Significantly Elevated

**Figure 2:**
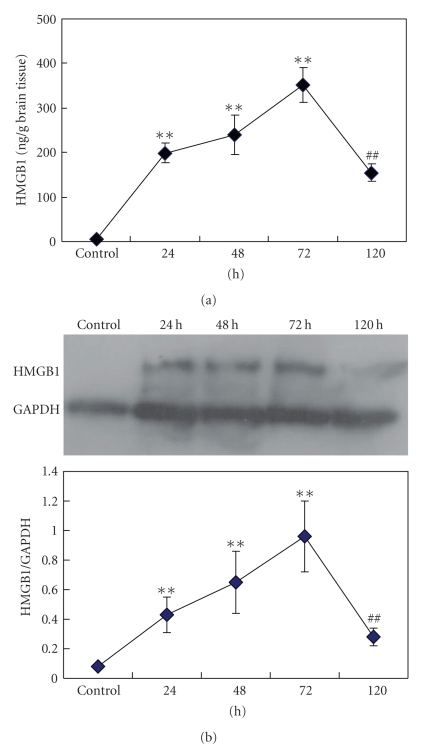
The level of HMGB1 increased significantly in the perihematomal tissue of ICH mice. Data are expressed as mean ± standard deviation. (a) ELISA assay of the changes of HMGB1 levels in the perihematomal tissue of ICH mice. (b) A representative picture of Western blot assay showing the changes of HMGB1 levels in the perihematomal tissue of mice. GAPDH was chosen as the internal control. The changes of HMGB1 were expressed as the ratio of the optical density values of HMGB1 band to the optical density values of GAPDH band. *n* = 15. **versus control; *P* < .01; ^##^versus 72 hours; *P* < .01.

Previous studies have established that acute ICH can lead to potent systemic inflammatory responses and production of large quantities of inflammatory factors. The findings above suggest that large amounts of HMGB1 are present in the peripheral blood of patients with acute ICH. Nevertheless, it remained to be determined whether the hematoma developed following ICH could stimulate the generation of HMGB1 in the perihematomal brain tissue. Accordingly, an ICH model was established in mice and changes in HMGB1 levels in the perihematomal brain tissue at various time points after ICH were examined with ELISA and Western blot assays. The results are shown in Figure 2. Compared with the control group, HMGB1 levels in the perihematomal brain tissue increased dramatically, peaked at 72 hours (352.4 ± 38.7 ng/mL), and started to decrease (154.2 ± 19.8 ng/mL) at 5 days. These results indicate that the hematoma developed following ICH stimulates the production of HMGB1 in the perihematomal brain tissue.

### 3.3. Heme Could Stimulate the Secretion of HMGB1 by Cultured Microglia

**Figure 3:**
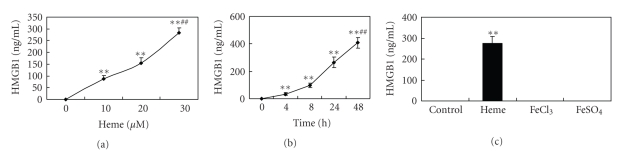
Heme could stimulate the secretion of HMGB1 by cultured microglia. Data are expressed as mean ± standard deviation, *n* = 6. (a) Microglia were cultured with various concentrations of heme (0 *μ*M, 10 *μ*M, 20 *μ*M, and 30 *μ*M) for 24 hours. Thereafter, the culture medium was replaced, and the cells were further cultured for 12 hours before collection of the supernatant for ELISA determination of HMGB1 levels. **versus 0 *μ*M; *P* < .01; ^##^versus 10 *μ*M, 20 *μ*M; *P* < .01. (b) Microglia were treated with 30 *μ*M of heme for various lengths of time (0 h, 4 h, 8 h, 24 h, and 48 h). Thereafter, the culture medium was replaced, and the cells were further cultured for another 12 hours before collection of the supernatant for ELISA determination of HMGB1 levels. **versus 0 h; *P* < .01; ^##^versus 4 h, 8 h, or 24 h; *P* < .01. (c) Microglia were treated with 30 *μ*M of heme, 30 *μ*M of FeCl_3_, or 30 *μ*M of FeSO_4_ for 24 hours. Thereafter, the culture medium was replaced, and the cells were further cultured for another 12 hours before collection of the supernatant for ELISA determination of HMGB1 levels. **versus control; *P* < .01.

Our results above demonstrated that the hematoma developed following ICH stimulated the production of HMGB1. However, it remained to be established what component in the hematoma stimulated brain tissues to secrete HMGB1. The main blood components, heme (hemoglobin) and Fe ions, are known to play an important role in ICH-caused neurological deficits [18]. In addition, a previous study showed that in some hemorrhagic diseases, blood components such as heme can cause tissue injury by stimulating macrophages to release inflammatory factors [17]. Since microglia are the major inflammatory cells in the brain, we examined whether heme and Fe^2+^/Fe^3+^, the two main blood components, could stimulate microglia to produce HMGB1. The results are shown in Figure 3. We found that heme could provoke significant production of HMGB1 from cultured microglia in a dose- and time-dependant manner. In contrast, both FeCl_3_ and FeSO_4_ failed to stimulate microglia to produce HMGB1. The above results suggest that heme in the hematoma after ICH can stimulate surrounding microglia to secrete HMGB1, leading to an increase in the HMGB1 level in the perihematomal brain tissue.

## 4. Discussion

To our knowledge, the present study is the first one to examine the early changes of HMGB1 following acute ICH. Our results showed that the serum level of HMGB1 increased significantly after acute ICH and was significantly positively correlated with stroke severity. Heme can stimulate notable release of HMGB1 from microglia. Hence, we speculate that the inflammatory factor HMGB1 may exert an important role in inflammation-caused neurological deficits after ICH. This molecule holds promise as a new early indicator for evaluating the severity and prognosis of ICH as well as a novel therapeutic target.

Previous studies have demonstrated that in the early stages after ICH, potent systemic inflammatory responses are elicited and large amounts of inflammatory factors are released, exacerbating the neurological impairment resulting from ICH. Our results also showed that the serum level of HMGB1 increased remarkably in the early stages following acute ICH (within 12 hours). However, the exact source of HMGB1 production following ICH remained to be determined whether it is produced in the peripheral blood, or it is generated due to the stimulation of the hematoma on surrounding brain tissues, or it is produced by both mechanisms? Our study found that HMGB1 levels in the perihematomal tissue in mice increased significantly after ICH, peaked at 72 hours, and started to drop at 5 days. These findings indicate that some components in the hematoma may stimulate surrounding tissues to produce HMGB1. Nevertheless, our results did not rule out the possibility that ICH itself can cause the activation of peripheral inflammatory cells which will subsequently produce HMGB1. Further studies into this issue are warranted. Our results also demonstrated that heme in the hematoma following ICH could induce microglia to secrete large quantities of HMGB1, whereas Fe^2+^/^3+^ ions did not have such a stimulatory effect. Therefore, we speculate that heme in the hematoma following ICH provokes the abundant production of HMGB1 from surrounding microglia. However, further studies are needed to determine the effect that HMGB1 will cause and the pathways that HMGB1 is involved in.

However, our study also has some limitations. First, the study was a single-center study including relatively few cases. Large-scale multicenter studies are needed to confirm the correlation between HMGB1 changes and the severity of ICH. Second, our study is only a preliminary investigation into the possible role of HMGB1 in ICH. More efforts are justified to identify the exact role and the underlying mechanism of HMGB1 involvement in the ICH-caused inflammatory injury.

## 5. Conclusion

The serum level of HMGB1 increases dramatically in patients with acute ICH and is significantly positively correlated with the severity of the stroke. Heme can stimulate significant production of HMGB1 from microglia. Our results suggest that HMGB1 may play an important role in the inflammatory injury resulting from ICH.

## Figures and Tables

**Table 1:** Clinical, biochemical, and radiological characteristics by subjects.

	Control (*n* = 41)	ICH		*P*-value
		Good outcome (mRS, 0–2), *n* = 31	Poor outcome (mRS, 3–6), *n* = 29	
Age, yr	63.2 (9)	65.4 (8)	67.4 (10)	NS
Sex (M/F, %)	61.4/	59.8	65.3	NS
Time from onset to admission, hr		6.5 (3.2–9.5)	5.9 (2.6–8.7)	NS
History of vascular risk factors (%)				
Hypertension	42.1	75.5**	72.6**	.0003
Diabetes	23.8	26.5	24.7	NS
Smoking habit (current)	16.5	11.7	15.3	NS
Alcohol consumption	18.7	21.6	26.8	NS
Prior medication with				
Lipid-lowering drugs	26.6	18.9	28.5	NS
Platelet inhibitors	33.0	28.6	31.4	NS
Vital signs				
Systolic blood pressure, mmHg		178 (32)	176 (28)	NS
Diastolic blood pressure, mmHg		101 (16)	98 (22)	NS
Maximal SBP in 24 hr, mmHg		198 (26)	192 (24)	NS
Maximal DBP in 24 hr, mmHg		110 (12)	108 (9)	NS
Body temperature, (°C)		36.5 (0.62)	36.2 (0.81)	NS
Glasgow scale at baseline		15 (14-15)	12 (10–14)	<.001
NIHSS at 10 days		9 (5–15)	21 (16–24)	<.001
mRS at 3 month		1.8 (0–2)	4 (3–6)	<.001
Neuroimaging findings				
Topography, % lobar		45.2	42.8	NS
ICH volume at baseline, mL		14.8 (6.8–22.0)	34.2 (15.0–52.4)	<.001
Edema volume at baseline, mL		3.2 (1.2–9.0)	12.0 (5.2–14.8)	<.01
ICH growth at 72 hours, ratio		0.08 (0.0–0.41)	0.11 (0.02–0.46)	NS
ICH growth at 7 days, ratio		−0.32 (−0.55–0.02)	0.01 (−0.19–0.29)	<.01
Edema growth at 72 hours, ratio		0.65 (0.03–4.32)	0.88 (0.46–3.34)	NS
Edema growth at 7 days, ratio		0.94 (0.00–3.10)	1.21 (0.12–2.54)	NS
Intraventricular bleeding, %		11.5	53.4	<.001
Mass effect, %		21.0	68.5	<.001
Laboratory parameters				
Serum glucose, mg/dL		125 (23)	117 (36)	NS
Platelet count, ×1000/mmc		226 (72)	241 (69)	NS
Leukocyte count, ×1000/mmc		7.8 (2.0)	8.1 (1.8)	NS
Plasma fibrinogen, mg/dL		455 (120)	447 (116)	NS
IL-6, pg/mL		102.6 (35.6)	184.2 (38.4)	<.01

TNF-*α*, pg/mL		54.3 (15.2)	95.3 (18.5)	<.05

Values are presented as proportions, mean (SD), or median (quartiles). CT was performed in 63 patients at 72 hours and 7 days. Reason to not perform followup CT were death in 2 patients at 72 hours. DBP: diastolic blood pressure; SBP: systolic blood pressure; NIHSS: National Institute of Health Stroke Scale; mRS: modified Rankin scale; mmc: cubic millimeter. **P < .01 versus control.
